# Hypertrophic Pyloric Stenosis in Infancy

**Published:** 1900-06-23

**Authors:** 


### HYPERTROPHIC PYLORIC STENOSIS IN INFANCY.

Dr. Eric Pritchard[1] draws attention to this rare condition. So far as he has been able to ascertain, the published records of fatal cases of hypertrophic pyloric stenosis occurring in infancy, which have been verified by post-mortem examinations, are 23 in number. After a careful review of the cases already published, Dr. Pritchard gives details of a case observed by himself. The child was apparently healthy at birth, was born at full time, of healthy parents, weighed nine pounds, and had one healthy elder brother living. It was fed by hand. During the first three weeks there were no symptoms except sweating. At the third week vomiting set in, the urine was very scanty, and the bowels constipated. Then the child persistently lost weight until at the seventh week it only weighed six pounds. At this time the emaciation was extreme; the tongue and fauces were remarkably clean; the abdomen was flaccid and carinated, and the vertebral column was easily palpated through the abdominal walls; there were no enlarged mesenteric glands; the upper part of the abdomen was more prominent and resonant to percussion; at times the outline of the stomach was quite evident and extended as far as, and below the umbilicus, and slow waves of contraction could be seen passing from the cardiac end to the pylorus, where, however, no tumour or sense of resistance could be made out. The temperature was normal, but during an attack of convulsions which afterwards occurred, the temperature rose to 104·5 deg. F. By aid of nutrient enemata and teaspoonful quantities of food, it seemed to put on a little weight, and vomiting ceased, but it died 12 days after it was first seen. At the autopsy the brain and meninges were slightly congested, and the lateral sinus was distended. The œsophagus was distended throughout, "the stomach was enlarged and capable of containing about seven ounces of water. The walls were slightly thickened, especially at the pyloric end. At the pylorus itself was a hard annular thickening, giving almost the appearance of the impaction of some foreign body about the size of a large filbert nut. A No. 5 catheter could just pass through the opening. On splitting the pylorus open the walls were found to be enormously thickened, and to consist of a pale homogeneous tissue." Microscopically there was nothing abnormal in this material. Dr. Pritchard suggests that in these cases the hypertrophy is secondary to over action of the sphincter, that the stenosis is chiefly due to spasm, and that the over action and the inco-ordinated contractions of the sphincter may be due to some fault in the nervous mechanism, although injudicious feeding may be a contributory factor.

[1] Archives of Pediatrics, April.

